# Cardiovascular Manifestations of Patients with Non-Alcoholic Fatty Liver Disease

**DOI:** 10.3390/metabo15030149

**Published:** 2025-02-23

**Authors:** Vlad Pădureanu, Mircea Cătălin Forțofoiu, Mircea Pîrșcoveanu, Rodica Pădureanu, Dumitru Rădulescu, Ionuț Donoiu, Denisa Floriana Vasilica Pîrșcoveanu

**Affiliations:** 1 Department of Internal Medicine, University of Medicine and Pharmacy Craiova, 200349 Craiova, Romania; vlad.padureanu@umfcv.ro (V.P.); catalin.fortofoiu@umfcv.ro (M.C.F.); 2 Department of Surgery, University of Medicine and Pharmacy Craiova, 200349 Craiova, Romania; mircea.pirscoveanu@umfcv.ro; 3 Department of Cardiology, University of Medicine and Pharmacy Craiova, 200349 Craiova, Romania; ionut.donoiu@umfcv.ro; 4 Department of Neurology, University of Medicine and Pharmacy Craiova, 200349 Craiova, Romania; denisa.pirscoveanu@umfcv.ro

**Keywords:** NAFLD, MAFLD, MASLD, cardiovascular disease, hypertension, atherosclerosis, arrythmias

## Abstract

**Background:** Non-alcoholic fatty liver disease (NAFLD), more recently redefined as metabolic-associated fatty liver disease (MAFLD), is now recognized as the most prevalent cause of chronic liver disease. Its strong association with cardiovascular disease (CVD) underscores its emerging role in global morbidity and mortality. **Objective:** This review critically examines the pathophysiological mechanisms that link NAFLD/MAFLD with CVD. It focuses on shared metabolic disturbances, inflammatory pathways, and alterations in the gut microbiota that contribute to hepatic and cardiovascular pathology. **Review and Gaps:** Current evidence highlights insulin resistance, dyslipidemia, systemic inflammation, and gut dysbiosis as pivotal factors connecting NAFLD/MAFLD to CVD. Despite these insights, inconsistencies in diagnostic criteria and a lack of validated non-invasive biomarkers hinder a clear understanding of the causal relationship between liver and cardiovascular diseases. **Conclusions:** Addressing these knowledge gaps through standardized diagnostic protocols and large-scale longitudinal studies is essential. Improved biomarker validation and clearer delineation of the underlying mechanisms will improve cardiovascular risk stratification and enable more personalized therapeutic strategies for patients with NAFLD/MAFLD.

## 1. Introduction

Non-alcoholic fatty liver disease (NAFLD) has become the most common cause of liver disease in the 21st century, with a prevalence ranging from 25% to 45% in the general adult population and up to 70% in people with type 2 diabetes [1,2,3,4,5]. It represents a spectrum of liver abnormalities that range from simple steatosis to non-alcoholic steatohepatitis (NASH), liver fibrosis, cirrhosis, and potentially hepatocellular carcinoma [3]. During the past decade, NAFLD has gained attention not only for its impact on liver-related morbidity but also for its significant extrahepatic manifestations, particularly its strong association with cardiovascular disease (CVD) [6,7].

The concept of NAFLD has recently undergone a redefinition, and the term metabolic-associated fatty liver disease (MAFLD) was proposed to reflect its intrinsic relationship with metabolic disorders, such as obesity, insulin resistance, dyslipidemia, and type 2 diabetes [8]. Unlike NAFLD, which was defined by the exclusion of alcohol consumption, MAFLD focuses on metabolic dysfunction as the primary criterion for diagnosis [9]. Although this new definition has provoked debate, many studies have acknowledged its potential to better capture the systemic nature of fatty liver disease and its cardiovascular impact [10].

The link between NAFLD and CVD is based on shared metabolic pathways, including insulin resistance, systemic inflammation, oxidative stress, and atherogenic dyslipidemia, which collectively increase the risk of subclinical and clinical cardiovascular events [11,12,13]. However, despite numerous studies, the causal relationship remains unclear. Some studies suggest that NAFLD contributes independently to CVD progression, while others argue that common metabolic risk factors mediate this association [14]. For example, a meta-analysis that included more than 5.8 million subjects and 99,668 cardiovascular events demonstrated that patients with NAFLD had a 64% increased risk of cardiovascular events, corresponding to a hazard ratio (HR) of 1.45 (95% CI: 1.31–1.61), compared to those without NAFLD [15].

An area of ongoing investigation is the role of liver inflammation in inducing vascular dysfunction and promoting a pro-atherogenic state. Studies have shown that NAFLD is associated with increased levels of inflammatory cytokines, such as IL-6 and TNF-α, which contribute to endothelial dysfunction and plaque instability [16,17]. Furthermore, intestinal dysbiosis, a hallmark of many metabolic disorders, has emerged as a potential mediator between NAFLD and cardiovascular risk. Dysbiosis leads to elevated levels of harmful metabolites like trimethylamine N-oxide (TMAO), which is linked to atherosclerosis [18].

In clinical practice, the lack of standardized non-invasive biomarkers to predict cardiovascular risk in patients with NAFLD remains a challenge. Although tools such as the NAFLD fibrosis score (NFS) and FIB-4 are commonly used to assess liver fibrosis, their predictive power for cardiovascular outcomes is still debated [19]. Therefore, addressing these understanding gaps is crucial for improving cardiovascular risk prediction and management in patients with NAFLD/MAFLD.

This review aims to provide a comprehensive overview of the pathophysiological mechanisms linking NAFLD/MAFLD with cardiovascular disease, focusing on shared metabolic pathways, key biomarkers, and potential areas for future research.

## 2. Synthesis of Data

**Figure 1:**
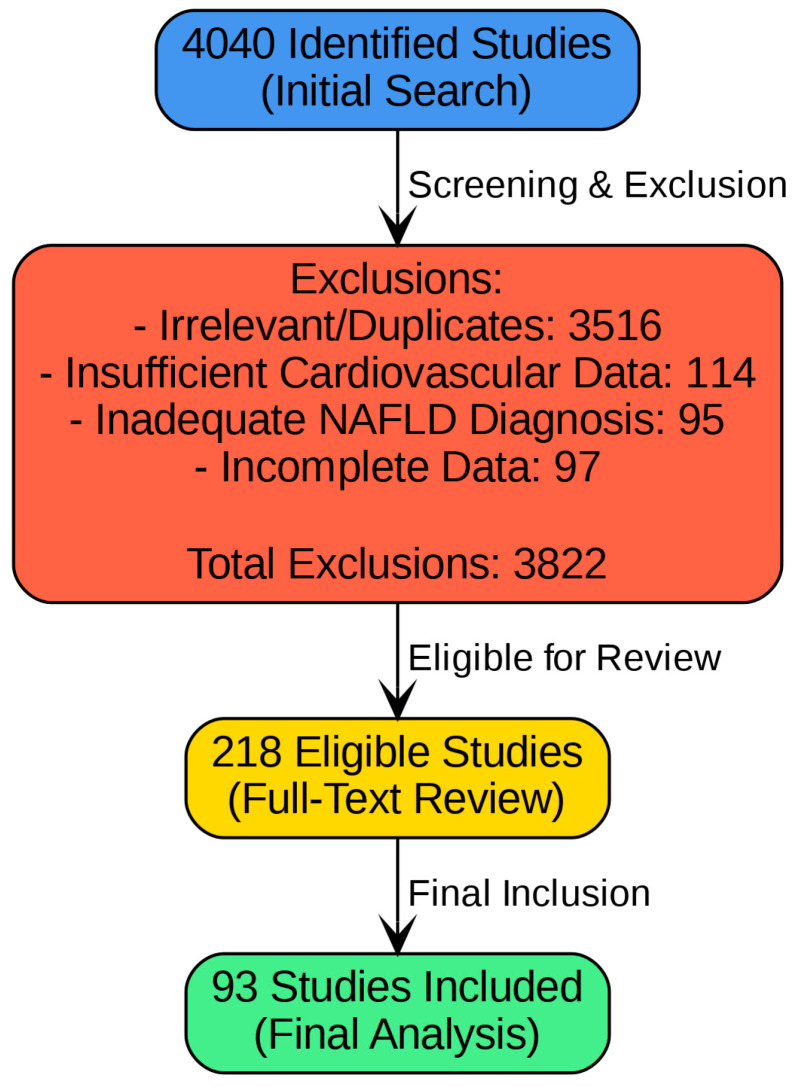
Study selection flowchart.

A detailed literature search was conducted using the PubMed and Scopus databases to identify relevant studies published between January 1990 and December 2020. The search included the following keywords: ‘NAFLD’, ‘MAFLD’, ‘MASLD’, ‘cardiovascular disease’, ‘atherosclerosis’, ‘hypertension’, and ‘arrhythmias’. The initial search yielded 3980 studies (Figure 1).

The inclusion criteria are as follows:

- Peer-reviewed articles addressing the association between NAFLD/MAFLD and cardiovascular disease;
- Studies evaluating cardiovascular outcomes and diagnosis of NAFLD using imaging, biopsy, or non-invasive methods;
- Full-text articles available in English.

The exclusion criteria are as follows:

- Non-English publications;
- Editorials, letters to the editor, conference abstracts, and review articles lacking original data;
- Studies with incomplete cardiovascular or diagnostic data.

Following the application of these criteria, the list was narrowed down to 218 relevant studies. A subsequent full-text evaluation resulted in the inclusion of 93 studies in the final review.

Although the PRISMA guidelines were not strictly followed, the selection process was structured to ensure thorough and in-depth coverage of the topic. The included studies span multiple methodologies, including cross-sectional studies, cohort studies, and systematic reviews, covering key mechanisms such as systemic inflammation, endothelial dysfunction, dyslipidemia, and the involvement of the gut microbiota.

## 3. Pathophysiology of Cardiovascular Involvement in NAFLD

### 3.1. Atherogenic Dyslipidemia and Lipid Metabolism

Atherogenic dyslipidemia is a hallmark of NAFLD and a key factor linking the disease to cardiovascular risk. Increased de novo hepatic lipogenesis and an increased rate of lipid uptake contribute to this dyslipidemic profile. The primary characteristics of atherogenic dyslipidemia include elevated intermediate-density lipoproteins (IDLs), low HDL cholesterol, small dense LDL particles, and elevated triglycerides [20].

Patients with NAFLD exhibit elevated levels of palmitic acid, a saturated fatty acid that integrates into very-low-density lipoproteins (VLDLs) and triggers vascular inflammation by activating toll-like receptors (TLRs) 2 and 4, a key mechanism linking lipid dysregulation with cardiovascular risk [21]. This lipid imbalance damages the endothelium and accelerates the formation atherosclerotic plaques through oxidative stress and inflammatory cascades. Furthermore, the ‘atherogenic’ lipid profile typical of NAFLD/MAFLD patients—marked by reduced levels of high-density lipoproteins (HDLs) and an abundance of small, dense low-density lipoprotein (LDL) particles—plays a central role in cholesterol deposition within arterial walls, enhancing plaque growth and instability [22,23].

### 3.2. Endothelial Dysfunction and Oxidative Stress

Endothelial dysfunction is a key mediator linking NAFLD to cardiovascular disease and is characterized primarily by impaired nitric oxide (NO) bioavailability, which compromises vascular homeostasis [24,25]. Both clinical studies and consensus guidelines underscore that an excess of reactive oxygen species (ROS) and lipoprotein-mediated inflammation generate significant oxidative stress, thereby accelerating NO degradation. For example, elevated levels of asymmetric dimethylarginine (ADMA), an endogenous inhibitor of NO synthase, have been consistently reported in patients with NAFLD, with some studies documenting increases of 30–40% compared to control groups, which in turn impair NO synthesis and contribute to endothelial dysfunction [26,27]. This cascade disrupts endothelial signaling, alters vascular tone, and shifts the balance toward sustained vasoconstriction, thus increasing the risk of hypertension, atherosclerosis, and other cardiovascular complications. In addition, hyperhomocysteinemia, often observed in NAFLD, further exacerbates endothelial dysfunction by reducing glutathione storage and amplifying oxidative damage [28,29]. Collectively, these mechanisms, supported by recent consensus statements, establish that ADMA accumulation and the concomitant pro-oxidative environment not only alter NO-mediated vasodilation but also foster a pro-thrombotic state, thus significantly increasing cardiovascular risk in patients with NAFLD.

**Figure 2:**
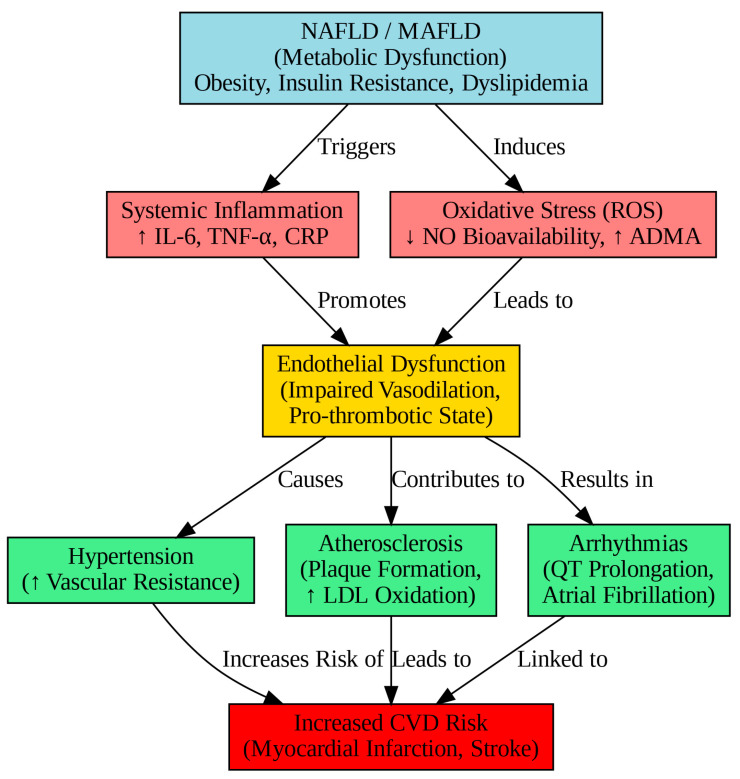
Pathophysiological mechanisms linking NAFLD/MAFLD to cardiovascular disease.

As illustrated in Figure 2, the pathophysiological cascade linking NAFLD/MAFLD to cardiovascular disease begins with a metabolic dysfunction that triggers systemic inflammation and oxidative stress, leading to endothelial dysfunction and ultimately resulting in clinical manifestations such as hypertension, atherosclerosis, and arrhythmias.

In addition, proinflammatory cytokines such as IL-6 and TNF-α, which are elevated in NAFLD, further alter endothelial function and destabilize atherosclerotic plaques, increasing the risk of acute cardiovascular events [30,31]. These mechanisms highlight how endothelial dysfunction bridges the gap between NAFLD and vascular complications.

### 3.3. Systemic Inflammation and Coagulation Abnormalities

Systemic inflammation is a key driver of cardiovascular complications in NAFLD, contributing to both early endothelial dysfunction and long-term vascular injury. Elevated inflammatory markers, including interleukin-6 (IL-6), tumor necrosis factor-alpha (TNF-α), and C-reactive protein (CRP), affect endothelial nitric oxide (NO) production, promoting vasoconstriction and reduced vascular elasticity. In addition, these proinflammatory cytokines exacerbate oxidative stress by enhancing the production of reactive oxygen species (ROS), leading to cumulative vascular damage and the accelerated development of atherosclerotic plaques [32,33]. This chronic inflammatory state promotes the production of acute phase proteins and activates the pathways involved in atherogenesis.

Patients with NAFLD often exhibit coagulation abnormalities due to an imbalance between procoagulant and anticoagulant factors, increasing their thrombotic risk. Elevated levels of coagulation factors, including factor VIII (FVIII), factor IX (FIX), factor XI (FXI), and fibrinogen, are commonly observed, enhancing clot formation. Meanwhile, natural anticoagulants such as protein C and antithrombin III are reduced, further tipping the balance toward hypercoagulability and increasing the likelihood of vascular complications [34,35]. This imbalance contributes to a hypercoagulable state, enhancing the risk of thrombus formation, myocardial infarction, and stroke. In addition, elevated levels of plasminogen activator inhibitor-1 (PAI-1) in patients with NAFLD contribute to a prothrombotic state by inhibiting fibrinolysis, the natural process of the body to break down blood clots. This inhibition increases the risk of persistent clot formation, vascular occlusion, and subsequent ischemic events, further amplifying cardiovascular risk [36].

### 3.4. Ectopic Fat Accumulation and Dysfunction of Adipose Tissue

Ectopic fat accumulation, particularly around the heart and liver, is closely linked to NAFLD and its cardiovascular complications. Epicardial adipose tissue, normally considered protective due to its anti-inflammatory and antifibrotic properties, undergoes phenotypic changes in the context of systemic inflammation associated with NAFLD [37,38].

As the phenotype of epicardial adipose tissue changes, it transforms into a proinflammatory environment, releasing cytokines and adipokines that promote fibrosis, inflammation, and cardiac dysfunction [39]. This contributes to left ventricular dysfunction, increased ventricular stiffness, and structural remodeling of the heart, which collectively increase the risk of heart failure. In addition, fat accumulation in the perivascular regions contributes to vascular inflammation, further exacerbating endothelial dysfunction.

### 3.5. Genetic Factors and Hepatokines

Genetic polymorphisms in the TM6SF2 and PNPLA3 genes are strongly associated with both the progression and severity of NAFLD, as well as its cardiovascular manifestations. Variants in these genes influence lipid metabolism, hepatocellular injury, and systemic inflammation, which exacerbate hepatic fat accumulation and promote vascular inflammation, atherosclerosis, and endothelial dysfunction. In a clinical study investigating genetic variants in the progression of NAFLD, the PNPLA3 and TM6SF2 mutations were shown to be significantly correlated with increased liver steatosis and fibrosis, confirming their role in disease progression. Such findings were supported by meta-analyses highlighting consistent associations between these genetic variants and cardiovascular risks, though the specific impact on cardiovascular outcomes may vary between cohorts of patients. [40]. These genetic variants predispose individuals to liver damage and metabolic dysregulation. Interestingly, some studies suggest that certain polymorphisms may exert cardioprotective effects in specific populations by modulating lipid metabolism and fat accumulation [41,42].

Hepatokines, liver-secreted proteins such as fetuin A, play a crucial role in linking NAFLD to cardiometabolic disease. Elevated levels of fetuin A have been associated with increased insulin resistance, systemic inflammation, and a higher incidence of cardiovascular events [43,44]. The interplay between genetic predisposition and hepatokine secretion highlights the multifactorial nature of cardiovascular risk in patients with NAFLD.

### 3.6. Gut Dysbiosis and Cardiovascular Risk

Gut dysbiosis, or an imbalance in gut microbiota, is another emerging factor linking NAFLD to cardiovascular disease [45]. Patients with NAFLD often exhibit intestinal microbiome imbalances, with significant alterations in taxonomic composition, including a reduction in beneficial bacteria such as Firmicutes and an overgrowth of harmful bacteria such as Escherichia coli and Ruminococcus. In a clinical study of fecal samples from 90 patients with NAFLD and 21 healthy controls using 16S rRNA gene sequencing, the findings revealed a decreased abundance of Bacteroidetes and Ruminococcaceae, along with an increased abundance of Lactobacillaceae, Veillonellaceae, and Dorea. Although changes in gut microbiota composition have been commonly reported in NAFLD studies, reproducibility issues remain, as some differences vary between study populations [46,47,48]. These imbalances contribute to increased intestinal permeability, endotoxemia, and systemic inflammation, which not only exacerbate liver damage and cardiovascular complications but also trigger changes that result in the elevated production of harmful metabolites, such as trimethylamine N-oxide (TMAO), known to promote foam cell formation and early atherosclerosis [17]. Furthermore, a compromised intestinal barrier allows for the translocation of microbial products such as lipopolysaccharides (LPSs) into the bloodstream, triggering systemic inflammation through the activation of toll-like receptors (TLRs) [49,50]. Modulation of the gut microbiome has thus been proposed as a potential therapeutic strategy to reduce cardiovascular risk in patients with NAFLD [51].

### 3.7. Atherosclerosis and Plaque Formation

Atherosclerosis, characterized by chronic inflammation and plaque build-up within arterial walls, is closely associated with NAFLD. The dyslipidemic profile of patients with NAFLD, marked by small dense LDL particles, elevated triglycerides, and reduced HDL levels, promotes the deposition of cholesterol and lipids within atherosclerotic plaques [21,22].

Inflammatory mediators produced by the liver, such as CRP and IL-6, further exacerbate plaque formation by promoting endothelial damage and the recruitment of macrophages to the plaque site [50]. NAFLD patients with advanced fibrosis or NASH have higher levels of acute-phase proteins, which contribute to plaque instability and rupture, increasing the risk of myocardial infarction and stroke [16,44].

## 4. Association Between CVD and NAFLD/MAFLD

Metabolic changes such as type 2 diabetes, obesity, dyslipidemia, and insulin resistance are more prevalent in patients with NAFLD, and their natural history explains both the elevated risk of CVDs and liver-related comorbidities. Given that natural history studies in communities with NAFLD have shown that CVDs are the leading cause of death in these groups, this is of significant clinical relevance [52,53]. In a similar vein, a population-based study that examined annual age-standardized extrahepatic mortality among NAFLD patients in the United States between 2007 and 2017 found that CVD was the most prevalent and increasing cause of death in NAFLD patients (around 20%) [12].

This association between NAFLD and CVD risk has been extensively explored in multiple population-based clinical studies, highlighting the role of subclinical cardiovascular changes.

NAFLD, as determined by the fatty liver index, was associated with the occurrence and development of carotid intima–media thickness (C-IMT), which predicts carotid plaques and CVD events. This study aimed to demonstrate that NAFLD is both a cause and an observer of metabolic syndrome and that steatosis plays a role in the onset of early atherosclerosis [54].

In a 2007 cross-sectional study conducted in Italy, Targher et al. examined the relationship between cardiovascular events and NAFLD in individuals with type 2 diabetes, going beyond subclinical cardiovascular changes to actual clinical outcomes. In their article, an ultrasound examination was used to identify NAFLD. Compared to patients without NAFLD, patients with NAFLD had significantly higher age- and sex-adjusted prevalences of peripheral (15.4% versus 10.0%), cerebrovascular (20.0% versus 13.3%), and coronary (26.6% versus 18.3%) vascular diseases; this association was valid even after controlling for sex, age, smoking, BMI, the duration of diabetes, HbA1C, LDL cholesterol, metabolic syndrome, and medications. These findings emphasize the strong association between NAFLD and cardiovascular events across various vascular sites [55].

The relationship between subclinical cardiovascular changes and NAFLD has also been the subject of numerous investigations. In a retrospective investigation, Pais and associates looked at the relationship between carotid atherosclerosis and NAFLD in a sizable group of French patients who were followed over time from 1995 to 2012.

Bonapace and associates concentrated on the relationship between NAFLD and cardiac changes in the context of diabetic patients. According to the existence and severity of steatosis, patients with type 2 diabetes specifically showed a higher prevalence of sub-clinical left ventricular diastolic dysfunction. This association, which was observed in diabetic patients with normal systolic function and without a history of ischemic heart disease, was unaffected by hypertension and a number of other variables, including sex, age, HbA1c, and triglycerides (TGs). The scientists proposed that NAFLD, a sign of ectopic fat buildup in the heart, was the cause of this connection [56].

The association between NAFLD and the incidence of CVD was further examined in a bigger meta-analysis conducted in 2021. About 5.8 million persons and 99,668 occurrences of both non-fatal and fatal (myocardial infarction, angina, hemorrhagic or ischemic strokes, or coronary revascularization procedures) CVD events were included in 36 longitudinal studies with a median follow-up of 6.5 years. A moderately elevated risk of either fatal or non-fatal CVD events was confirmed to be linked to NAFLD (pooled random effects HR: 1.45; 95% CI: 1.31–1.61; I2 = 86.18%). Interestingly, the risk persisted even after controlling for preexisting diabetes, smoking, age, sex, adiposity metrics, and other cardiometabolic risk variables. The risk significantly increased with the severity of NAFLD, as determined by ultrasonography plus elevated serum GGT, ultrasonographic scores, increased 18F-fluorodeoxyglucose (FDG) uptake on PET, or the severity of liver fibrosis as determined by histology or NFS, according to substantial evidence from this meta-analysis [57].

Cross-sectional studies evaluating subclinical cardiovascular changes have demonstrated that the severity of NAFLD is associated with an increase in carotid intima–media thickness and the prevalence of carotid plaques. For example, a retrospective analysis involving more than 85,000 subjects found that NAFLD was associated with a 60% higher likelihood of forming carotid plaques (OR: 1.60; 95% CI: 1.45–1.78) [50]. In a retrospective cohort study, Sinn et al. studied the relationship between the progression of coronary atherosclerosis, as determined by CT, and NAFLD, which was identified through ultrasonound. Patients with NAFLD at baseline experienced a faster rate of progression of coronary atherosclerosis than those without NAFLD, and their risk increased further when their fibrosis was assessed using non-invasive scoring [58]. In clinical practice, the lack of standardized non-invasive biomarkers for predicting cardiovascular risk in NAFLD patients remains a challenge. Tools such as the NAFLD fibrosis score (NFS) and FIB-4 are commonly used to assess liver fibrosis, but their predictive accuracy for cardiovascular outcomes remains inconsistent [59]. These limitations highlight the need for improved biomarker validation and the integration of cardiovascular screening in the management of NAFLD. Instead, an Italian study linked biopsy-proven NAFLD to changes in the morphology and function of the cardiovascular system. Compared to those with milder fibrosis (F0–F2), those with advanced fibrosis (F3–F4) showed more epicardial fat.

Additionally, severe liver fibrosis was associated with several echocardiographic indicators, including left ventricular mass, diastolic posterior wall thickness, ejection fraction (EF), relative wall thickness, and left atrial volume [60]. In a cross-sectional investigation, Zhang and colleagues examined the renal and cardiovascular burden of an illness in persons with MAFLD and NAFLD in order to compare the two conditions. The prevalence and total number of MAFLD patients rose dramatically and exceeded those of NAFLD cases, according to nine continuous studies conducted over an 18-year period from 1999 to 2016. Compared to the non-MAFLD group, the MAFLD group had significantly higher odds for all three components of the metabolic syndrome: hypertension, diabetes, dyslipidemia, and obesity. This was particularly true for central obesity (OR = 17.05, 95% CI: 15.32–18.97) and diabetes (OR = 5.73, 95% CI: 5.10–6.45). Furthermore, the Framingham cardiovascular score of the NAFLD group was lower than that of the MAFLD group (OR = 3.2, 95% CI: 2.8–3.6 versus OR = 3.7, 95% CI: 3.4–4.1), and patients with MAFLD had a significantly higher 10-year CVD risk of stroke and myocardial infarction.

According to Zhang et al. [61], MAFLD may have a higher absolute cardiorenal burden than NAFLD in their investigation. In light of all of these data, international guidelines recommend that all patients with cardiometabolic diseases be examined for NAFLD/MAFLD and its severity, as well as that all patients with NAFLD be screened for CV risk irrespective of traditional CV risk factors [62].

Despite numerous studies that establish a strong association between NAFLD and cardiovascular disease, it is unclear whether this relationship is causal or primarily mediated by common metabolic risk factors. Addressing this gap requires longitudinal cohort studies and clinical trials focused on disentangling the direct impact of liver inflammation versus metabolic syndrome on cardiovascular outcomes [15]. In addition, incorporating microbiome-based interventions could provide new therapeutic pathways to mitigate cardiovascular risk.

### 4.1. Atherosclerosis and NAFLD

Atherosclerosis is directly related to acute coronary syndrome and stroke and is characterized by the formation of neo-intimal cholesterotic plaques in the major arteries. Numerous studies have shown that NAFLD is associated with a higher prevalence of ischemic heart disease because it is a risk factor for atherosclerosis [63,64]. Subclinical indicators of atherosclerosis, such as the CAC score [61,65], carotid intima–media thickness (cIMT) [66,67], or arterial stiffness measured by the brachial–ankle index, have been used to validate the extensive documentation of atherosclerosis in patients with NAFLD. Even among patients with normal BMI, prospective studies have shown that patients with NAFLD have higher CAC scores than those without NAFLD [68,69].

Regardless of obesity, dyslipidemia, or type 2 diabetes, patients with NAFLD had greater cIMT and annual rates of CAC advancement [61,70,71]. Additionally, elevated cIMT was linked to liver fibrosis as determined by aspartate transaminase to platelet ratio index (APRI) scores and fibrosis-4 (FIB4) [72]. NAFLD is associated with the development of plaques in the carotid, iliac, or celiac trunk arteries in addition to the coronary arteries [73], as well as a propensity to multiarterial calcifications. Furthermore, NAFLD has been associated with unstable coronary plaques [74] and endothelial dysfunction [75], which explains why these individuals are at a significant risk of ischemic events [76]. Additionally, when NAFLD was present, patients with STEMI had a worse long-term prognosis and a greater short-term death rate [77]. Subclinical atherosclerosis was substantially more common in patients with NAFLD (OR = 1.60, 95% CI: 1.45–1.78), according to a meta-analysis involving more than 85,000 patients [78]. In addition to cardiovascular risk factors such as obesity, dyslipidemia, arterial hypertension, and type 2 diabetes, NAFLD increases the risk of atherosclerosis and predisposes individuals to the formation of unstable plaques.

### 4.2. Cardiac Structure and NAFLD

Structural heart disease is associated with NAFLD. In individuals with NAFLD, diastolic dysfunction and heart failure with a maintained ejection fraction and enhanced myocardial remodeling are common findings [33]. These alterations can cause arrhythmias and an increased risk of CVD events [79], in addition to an elevated risk of aortic sclerosis [80].

### 4.3. Arrhythmias and NAFLD

The prolonged QTc interval [81] and an elevated incidence of atrial fibrillation [82] have been associated with NAFLD. The physiopathological processes that cause arrhythmias in NAFLD patients include the development of cardiac fibrosis after an increase in proinflammatory adipocytokines linked to an increase in epicardial obesity [33]. Targher et al. reported that NAFLD is independently associated with an increased risk of atrial fibrillation. In their prospective study on patients with type 2 diabetes, the presence of NAFLD was associated with a higher risk of atrial fibrillation, with an odds ratio of 4.49 (95% CI: 1.6–12.9), even after adjusting for traditional cardiovascular risk factors [82].

With an adjusted OR of 1.88 for a 95% CI between 1.03 and 3.45, a different prospective study with patients followed for 16.3 years found an independent link between atrial fibrillation and NAFLD [83]. In a retrospective analysis of type 2 diabetic patients who had 24 h Holter monitoring, ventricular arrhythmias were also associated with NAFLD. The authors reported an OR of 3.01 for a 95% CI between 1.26 and 7.17 after controlling for covariates [84].

### 4.4. NAFLD and Hypertension

The complete explanation of the relationship between NAFLD and hypertension is still pending. There are signs that NAFLD-related systemic inflammation can promote the activation of the sympathetic nervous system, which could lead to hypertension [85]. In addition, insulin resistance can increase blood pressure by increasing free fatty acids, which result in perivascular fat deposits near the veins and the renal sinus. In addition, gut dysbiosis and elevated homocysteine levels associated with NAFLD can increase oxidative stress and hence promote hypertension [86]. Given the high prevalence of comorbid diabetes and hypertension in NAFLD patients, the combined effects of these conditions significantly elevate the risk of stroke and other cardiovascular events. Studies in sub-Saharan Africa have shown that people with both conditions are at much higher risk of stroke, highlighting the importance of strict management of blood pressure and blood sugar levels to mitigate these risks [87]. There was significant variation in the criteria used to diagnose NAFLD, despite the fact that multiple investigations showed a correlation between the disease and hypertension [88,89].

Although some studies used MRI [89] or surrogate scores such as FLI [88], others used ultrasonography [89,90] to diagnose NAFLD. In a prospective study involving 3191 German patients, NAFLD was significantly associated with an increased risk of hypertension, with an odds ratio of 3.1 (95% CI: 1.7–5.8). This association highlights the strong link between liver steatosis and elevated blood pressure, independent of other metabolic factors [89]. Patients with NAFLD were found to have a higher risk of prehypertension in a larger prospective study conducted in South Korea, which included 11,350 male patients. It is interesting to note that the risk changed with the severity of NAFLD [91]. Huh et al. conducted a prospective longitudinal study of 1521 participants without cardiovascular disease to assess the risk for hypertension. The authors discovered that the NAFLD group had a higher risk of hypertension, as determined via FLI, and that the risk grew progressively in line with the FLI value [91]. More recently, Lorbeer et al. found a correlation between liver fat content, high blood pressure, and increased risks of hypertension in a research that measured the liver fat fraction using MRI [90].

## 5. Limitations

This review is subject to several limitations. First, there is significant heterogeneity in NAFLD diagnostic criteria, imaging modalities, and non-invasive markers used in all studies, which may introduce inconsistencies in the findings. Furthermore, most of the included studies are observational or cross-sectional, making it difficult to establish a definitive causal relationship between NAFLD and cardiovascular outcomes. Variability in study populations, geographic regions, and methodological approaches further limits the generalizability of the results. Finally, reproducibility issues, particularly in emerging areas such as gut microbiota research, highlight the need for standardized protocols and larger prospective cohort studies to better delineate these complex associations.

## 6. Conclusions

NAFLD, now more accurately defined as MAFLD in light of its metabolic underpinnings, is strongly associated with an increased risk of cardiovascular disease. The evidence reviewed here demonstrates that NAFLD/MAFLD is linked to various adverse cardiovascular outcomes, including hypertension, atherosclerosis, and arrhythmias, through shared mechanisms such as insulin resistance, systemic inflammation, dyslipidemia, and oxidative stress. Although the severity of NAFLD is correlated with worse cardiovascular outcomes, more longitudinal research is essential to unravel the causal pathways involved. The standardization of diagnostic criteria and the validation of non-invasive biomarkers will be critical for improving cardiovascular risk stratification and enabling more targeted therapeutic interventions for patients with NAFLD/MAFLD.

Given these gaps, clinicians should prioritize metabolic risk management in NAFLD patients through glycemic control, weight loss, and the treatment of hyperlipidemia and hypertension. Hepatologists and cardiologists must collaborate within a multidisciplinary framework, recognizing that progressive NAFLD significantly increases cardiovascular risk and that CVD patients may also exhibit advanced liver disease. Addressing these issues through targeted interventions and improved screening strategies is essential to reduce the burden of CVD in this population.

## Data Availability

Not applicable.

## References

[1] Younossi Z.M. (2019). Non-alcoholic fatty liver disease—A global public health perspective. J. Hepatol..

[2] Perumpail B.J., Khan M.A., Yoo E.R., Cholankeril G., Kim D., Ahmed A. (2017). Clinical epidemiology and disease burden of nonalcoholic fatty liver disease. World J. Gastroenterol..

[3] Bertot L.C., Adams L. (2016). The natural course of non-alcoholic fatty liver disease. Int. J. Mol. Sci..

[4] Matteoni C.A., Younossi Z.M., Gramlich T., Boparai N., Liu Y.C., McCullough A.J. (1999). Nonalcoholic fatty liver disease: A spectrum of clinical and pathological severity. Gastroenterology.

[5] Leite N.C., Salles G.F., Araujo A.L., Villela-Nogueira C.A., Cardoso C.R. (2009). Prevalence and associated factors of non-alcoholic fatty liver disease in patients with type-2 diabetes mellitus. Liver Int..

[6] Assy N., Kaita K., Mymin D., Levy C., Rosser B., Minuk G. (2000). Fatty infiltration of liver in hyperlipidemic patients. Dig. Dis. Sci..

[7] Li A.A., Ahmed A., Kim D. (2020). Extrahepatic manifestations of non-alcoholic fatty liver disease. Gut Liver.

[8] Pădureanu V., Dop D., Drăgoescu A.N., Pădureanu R., Mușetescu A.E., Nedelcu L. (2021). Non-alcoholic fatty liver disease and hematologic manifestations (Review). Exp. Ther. Med..

[9] Adams L.A., Anstee Q.M., Tilg H., Targher G. (2017). Non-alcoholic fatty liver disease and its relationship with cardiovascular disease and other extrahepatic diseases. Gut.

[10] Angulo P., Kleiner D.E., Dam-Larsen S., Adams L.A., Bjornsson E.S., Charatcharoenwitthaya P., Mills P.R., Keach J.C., Lafferty H.D., Stahler A. (2015). Liver fibrosis, but no other histologic features, is associated with long-term outcomes of patients with nonalcoholic fatty liver disease. Gastroenterology.

[11] Ekstedt M., Hagström H., Nasr P., Fredrikson M., Stål P., Kechagias S., Hultcrantz R. (2015). Fibrosis stage is the strongest predictor for disease-specific mortality in NAFLD after up to 33 years of follow-up. Hepatology.

[12] Kim D., Adejumo A.C., Yoo E.R., Iqbal U., Li A.A., Pham E.A., Cholankeril G., Glenn J.S., Ahmed A. (2019). Trends in mortality from extrahepatic complications in patients with chronic liver disease, from 2007 through 2017. Gastroenterology.

[13] Baratta F., Pastori D., Angelico F., Balla A., Paganini A.M., Cocomello N., Ferro D., Violi F., Sanyal A.J., Del Ben M. (2020). Nonalcoholic fatty liver disease and fibrosis associated with increased risk of cardiovascular events in a prospective study. Clin. Gastroenterol. Hepatology.

[14] Brouwers M.C.G.J., Simons N., Stehouwer C.D.A., Isaacs A. (2020). Non-alcoholic fatty liver disease and cardiovascular disease: Assessing the evidence for causality. Diabetologia.

[15] Targher G., Byrne C.D., Lonardo A., Zoppini G., Barbui C. (2016). Non-alcoholic fatty liver disease and risk of incident cardiovascular disease: A meta-analysis. J. Hepatol..

[16] Pipitone R.M., Ciccioli C., Infantino G., La Mantia C., Parisi S., Tulone A., Pennisi G., Grimaudo S., Petta S. (2023). MAFLD: A Multisystem Disease. Ther. Adv. Endocrinol. Metab..

[17] Eslam M., Newsome P.N., Sarin S.K., Anstee Q.M., Targher G., Romero-Gomez M., Zelber-Sagi S., Wai-Sun Wong V., Dufour J.-F., Schattenberg J.M. (2020). A New Definition for Metabolic Dysfunction-Associated Fatty Liver Disease: An International Expert Consensus Statement. J. Hepatol..

[18] Grabherr F., Grander C., Effenberger M., Schwärzler J., Tilg H. (2022). MAFLD: What 2 Years of the Redefinition of Fatty Liver Disease Has Taught Us. Ther. Adv. Endocrinol. Metab..

[19] Chan K.E., Koh T.J.L., Tang A.S.P., Quek J., Yong J.N., Tay P., Tan D.J.H., Lim W.H., Lin S.Y., Huang D. (2022). Global prevalence and clinical characteristics of metabolic-associated fatty liver disease: A meta-analysis and systematic review of 10 739 607 individuals. J. Clin. Endoc. Metab..

[20] Liu J., Ayada I., Zhang X., Wang L., Li Y., Wen T., Ma Z., Bruno M.J., de Knegt R.J., Cao W. (2022). Estimating global prevalence of metabolic dysfunction-associated fatty liver disease in overweight or obese adults. Clin. Gastroenterol. Hepatol..

[21] Korenblat K.M., Fabbrini E., Mohammed B.S., Klein S. (2008). Liver, muscle, and adipose tissue insulin action is directly related to intrahepatic triglyceride content in obese subjects. Gastroenterology.

[22] Stahl E.P., Dhindsa D.S., Lee S.K., Sandesara P.B., Chalasani N.P., Sperling L.S. (2019). Nonalcoholic fatty liver disease and the heart. J. Am. Coll. Cardiol..

[23] Boren J., Chapman M.J., Krauss R.M., Packard C.J., Bentzon J.F., Binder C.J., Daemen M.J., Demer L.L., Hegele R.A., Nicholls S.J. (2020). Low-density lipoproteins cause atherosclerotic cardiovascular disease: Pathophysiological, genetic, and therapeutic insights: A consensus statement from the European Atherosclerosis Society Consensus Panel. Eur. Heart J..

[24] Lechner K., McKenzie A.L., Krankel N., Von Schacky C., Worm N., Nixdorff U., Lechner B., Scherr J., Weingärtner O., Krauss R.M. (2020). High-risk atherosclerosis and metabolic phenotype: The roles of ectopic adiposity, atherogenic dyslipidemia, and inflammation. Metab. Syndr. Relat. Disord..

[25] Francque S.M., van der Graaff D., Kwanten W.J. (2016). Nonalcoholic fatty liver disease and cardiovascular risk: Pathophysiological mechanisms and implications. J. Hepatol..

[26] Scholz G.H., Hanefeld M. (2016). Metabolic vascular syndrome: New insights into a multidimensional network of risk factors and diseases. Visc. Med..

[27] de Carvalho S.C.R., Muniz M.T.C., Siqueira M.D.V., Siqueira E.R.F., Gomes A.V., Silva K.A., Bezerra L.C.L., D’Almeida V., de Oliveira C.P.M.S., Beltrão Pereira L.M. (2013). M. Plasmatic higher levels of homocysteine in Non-alcoholic fatty liver disease (NAFLD). Nutr. J..

[28] Pastore A., Alisi A., di Giovamberardino G., Crudele A., Ceccarelli S., Panera N., Dionisi-Vici C., Nobili V. (2014). Plasma levels of homocysteine and cysteine increased in pediatric NAFLD and strongly correlated with severity of liver damage. Int. J. Mol. Sci..

[29] Tripodi A., Fracanzani A.L., Primignani M., Chantarangkul V., Clerici M., Mannucci P.M., Peyvandi F., Bertelli C., Valenti L., Fargion S. (2014). Procoagulant imbalance in patients with non-alcoholic fatty liver disease. J. Hepatol..

[30] Meex R.C.R., Watt M.J. (2017). Hepatokines: Linking non-alcoholic fatty liver disease and insulin resistance. Nat. Rev. Endocrinol..

[31] Stefan N., Haring H.-U. (2013). The role of hepatokines in metabolism. Nat. Rev. Endocrinol..

[32] Despres J.-P. (2012). Body fat distribution and risk of cardiovascular disease. Circulation.

[33] Packer M. (2018). Epicardial adipose tissue may mediate deleterious effects of obesity and inflammation on the myocardi-um. J. Am. Coll. Cardiol..

[34] Tsaban G., Wolak A., Avni-Hassid H., Gepner Y., Shelef I., Henkin Y., Schwarzfuchs D., Cohen N., Bril N., Rein M. (2017). Dynamics of intrapericardial and extrapericardial fat tissues during longterm, dietary-induced, moderate weight loss. Am. J. Clin. Nutr..

[35] Dongiovanni P., Petta S., Maglio C., Fracanzani A.L., Pipitone R., Mozzi E., Motta B.M., Kaminska D., Rametta R., Grimaudo S. (2015). Transmembrane 6 superfamily member 2 gene variant disentangles non-alcoholic steatohepatitis from cardiovascular disease. Hepatology.

[36] Lauridsen B.K., Stender S., Kristensen T.S., Kofoed K.F., Køber L., Nordestgaard B.G., Tybjærg-Hansen A. (2018). Liver fat content, non-alcoholic fatty liver disease, and ischaemic heart disease: Mendelian randomization and meta-analysis of 279 013 individuals. Eur. Heart J..

[37] Aron-Wisnewsky J., Vigliotti C., Witjes J., Le P., Holleboom A.G., Verheij J., Nieuwdorp M., Clément K. (2020). Gut microbiota and human NAFLD: Disentangling microbial signatures from metabolic disorders. Nat. Rev. Gastroenterol. Hepatol..

[38] Demir M., Lang S., Martin A., Farowski F., Wisplinghoff H., Vehreschild M.J.G.T., Krawczyk M., Nowag A., Scholz C.J., Kretzschmar A. (2020). Phenotyping non-alcoholic fatty liver disease by the gut microbiota: Ready for prime time?. J. Gastroenterol. Hepatol..

[39] Ma J., Li H. (2018). The role of gut microbiota in atherosclerosis and hypertension. Front. Pharmacol..

[40] Tang W.H.W., Backhed F., Landmesser U., Hazen S.L. (2019). Intestinal microbiota in cardiovascular health and disease. J. Am. Coll. Cardiol..

[41] Caussy C., Tripathi A., Humphrey G., Bassirian S., Singh S., Faulkner C., Bettencourt R., Rizo E., Richards L., Zhenjiang Z Xu Z.Z. (2019). A gut microbiome signature for cirrhosis due to nonalcoholic fatty liver disease. Nat. Commun..

[42] Del Chierico F., Nobili V., Vernocchi P., Russo A., De Stefanis C., Gnani D., Furlanello C., Zandonà A., Paci P., Capuani G. (2017). Gut microbiota pro:ling of pediatric nonalcoholic fatty liver disease and obese patients unveiled by an integrated metaomics-based approach. Hepatology.

[43] Da Silva H.E., Teterina A., Comelli E.M., Taibi A., Arendt B.M., Fischer S.E., Lou W., Johane P Allard J.P. (2018). Nonalcoholic fatty liver disease is associated with dysbiosis independent of body mass index and insulin resistance. Sci. Rep..

[44] Koeth R.A., Lam-Galvez B.R., Kirsop J., Wang Z., Levison B.S., Gu X., Copeland M.F., Bartlett D., Cody D.B., Dai H.J. (2018). l-Carnitine in omnivorous diets induces an atherogenic gut microbial pathway in humans. J. Clin. Investig..

[45] Ormazabal V., Nair S., Elfeky O., Aguayo C., Salomon C., Zuñiga F.A. (2018). Association between insulin resistance and the development of cardiovascular disease. Cardiovasc. Diabetol..

[46] Siddiqui M.S., Fuchs M., Idowu M.O., Luketic V.A., Boyett S., Sargeant C., Stravitz R.T., Puri P., Matherly S., Richard K Sterling R.K. (2015). Severity of nonalcoholic fatty liver disease and progression to cirrhosis are associated with atherogenic lipoprotein profile. Clin. Gastroenterol. Hepatol..

[47] Vekic J., Zeljkovic A., Cicero A.F.G., Janez A., Pantea Stoian A., Sonmez A., Rizzo M. (2022). Atherosclerosis development and progression: The role of atherogenic small, dense LDL. Medicina.

[48] Targher G., Corey K.E., Byrne C.D. (2021). NAFLD, and cardiovascular and cardiac diseases: Factors influencing risk, prediction and treatment. Diabetes Metab..

[49] Shoelson S.E., Lee J., Goldfine A.B. (2006). Inflammation and insulin resistance. J. Clin. Investig..

[50] Targher G., Day C.P., Bonora E. (2010). Risk of cardiovascular disease in patients with nonalcoholic fatty liver disease. N. Engl. J. Med..

[51] Ajmal M.R., Yaccha M., Malik M.A., Rabbani M.U., Ahmad I., Isalm N., Abdali N. (2014). Prevalence of nonalcoholic fatty liver disease (NAFLD) in patients of cardiovascular diseases and its association with hs-CRP and TNF-α. Indian Heart J..

[52] Ozturk K., Kurt O., Dogan T., Ozen A., Demirci H., Yesildal F., Kantarcioglu M., Turker T., Guler A.K., Karslioglu Y. (2016). Pentraxin 3 is a predictor for fibrosis and arterial stiffness in patients with nonalcoholic fatty liver disease. Gastroenterol. Res. Pract..

[53] Aron-Wisnewsky J., Clément K. (2015). The gut microbiome, diet, and links to cardiometabolic and chronic disorders. Nat. Rev. Nephrol..

[54] European Association for the Study of the Liver (EASL), European Association for the Study of Diabetes (EASD), European Association for the Study of Obesity (EASO) (2016). EASL-EASD-EASO clinical practice guidelines for the management of non-alcoholic fatty liver disease. Obes. Facts..

[55] Targher G., Bertolini L., Padovani R., Rodella S., Tessari R., Zenari L., Day C., Arcaro G. (2007). Prevalence of nonalcoholic fatty liver disease and its association with cardiovascular disease among type 2 diabetic patients. Diabetes Care.

[56] Bonapace S., Perseghin G., Molon G., Canali G., Bertolini L., Zoppini G., Barbieri E., Targher G. (2012). Nonalcoholic fatty liver disease is associated with left ventricular diastolic dysfunction in patients with type 2 diabetes. Diabetes Care.

[57] Kim D., Kim W.R., Kim H.J., Therneau T.M. (2013). Association between non invasive fibrosis markers and mortality among adults with nonalcoholic fatty liver disease in the United States. Hepatology.

[58] Sinn D.H., Kang D., Chang Y., Ryu S., Gu S., Kim H., Seong D., Cho S.J., Yi B.K., Hyung-Doo Park H.D. (2017). Non-alcoholic fatty liver disease and progression of coronary artery calcium score: A retrospective cohort study. Gut.

[59] Pais R., Giral P., Khan J.F., Rosenbaum D., Housset C., Poynard T., Ratziu V., LIDO Study Group (2016). Fatty liver is an independent predictor of early carotid atherosclerosis. J. Hepatol..

[60] Mantovani A., Csermely A., Petracca G., Beatrice G., Corey K.E., Simon T.G., Byrne C.D., Targher G. (2021). Non-alcoholic fatty liver disease and risk of fatal and non-fatal cardiovascular events: An updated systematic review and meta-analysis. Lancet Gastroenterol. Hepatol..

[61] Zhang H.J., Wang Y.Y., Chen C., Lu Y.L., Wang N.J. (2021). Cardiovascular and renal burdens of metabolic associated fatty liver disease from serial US national surveys, 1999–2016. Chin. Med. J..

[62] Petta S., Argano C., Colomba D., Cammà C., Di Marco V., Cabibi D., Tuttolomondo A., Marchesini G., Pinto A., Licata G. (2015). Epicardial fat, cardiac geometry and cardiac function in patients with non-alcoholic fatty liver disease:association with the severity of liver disease. J. Hepatol..

[63] European Association for the Study of the Liver (2021). EASL clinical practice guidelines on non-invasive tests for evaluation of liver disease severity and prognosis—2021 update. J. Hepatol..

[64] Musso G., Gambino R., Cassader M., Pagano G. (2011). Metaanalysis: Natural history of non-alcoholic fatty liver disease (NAFLD) and diagnostic accuracy of non-invasive tests for liver disease severity. Ann. Med..

[65] Stepanova M., Younossi Z.M. (2012). Independent association between nonalcoholic fatty liver disease and cardiovascular disease in the US population. Clin. Gastroenterol. Hepatol..

[66] Cho Y.K., Kang Y.M., Yoo J.H., Lee J., Lee S.E., Yang D.H., Kang J.W., Park J.Y., Jung C.H., Hong-Kyu Kim H.K. (2018). The impact of non-alcoholic fatty liver disease and metabolic syndrome on the progression of coronary artery calcification. Sci. Rep..

[67] Gill C., Vatcheva K.P., Pan J.-J., Smulevitz B., McPherson D.D., Fallon M., McCormick J.B., Fisher-Hoch S.P., Laing S.T. (2017). Frequency of non-alcoholic fatty liver disease and subclinical atherosclerosis among young Mexican Americans. Am. J. Cardiol..

[68] Zhou Y.Y., Zhou X.D., Wu S.J., Fan D.H., Van Poucke S., Chen Y.P., Fu S.W., Zheng M.H. (2018). Nonalcoholic fatty liver disease contributes to subclinical atherosclerosis: A systematic review and meta-analysis. Hepatol. Commun..

[69] Chang Y., Ryu S., Sung K.-C., Cho Y.K., Sung E., Kim H.-N., Jung H.-S., Yun K.E., Ahn J., Shin H. (2019). Alcoholic and non-alcoholic fatty liver disease and associations with coronary artery calcification: Evidence from the Kangbuk Samsung Health Study. Gut.

[70] Oni E., Budo M.J., Zeb I., Li D., Veledar E., Polak J.F., Blankstein R., Wong N.D., Blaha M.J., Agatston A. (2019). Nonalcoholic fatty liver disease is associated with arterial distensibility and carotid intima-media thickness: (from the multi-ethnic study of atherosclerosis). Am. J. Cardiol..

[71] Zheng R., Du Z., Wang M., Mao Y., Mao W. (2018). A longitudinal epidemiological study on the triglyceride and glucose index and the incident nonalcoholic fatty liver disease. Lipids Health Dis..

[72] Zheng J., Zhou Y., Zhang K., Qi Y., An S., Wang S., Zhao X., Tang Y.-D. (2018). Association between nonalcoholic fatty liver disease and subclinical atherosclerosis: A cross-sectional study on population over 40 years old. BMC Cardiovasc. Disord..

[73] Xin Z., Zhu Y., Wang S., Liu S., Xu M., Wang T., Lu J., Chen Y., Zhao Z., Wang W. (2020). Associations of subclinical atherosclerosis with nonalcoholic fatty liver disease and fibrosis assessed by non-invasive score. Liver Int..

[74] Koo B.K., Allison M.A., Criqui M.H., Denenberg J.O., Wright C.M. (2020). The association between liver fat and systemic calcified atherosclerosis. J. Vasc. Surg..

[75] Narayan J., Das H.S., Nath P., Singh A., Mishra D., Padhi P.K., Singh S.P. (2020). Endothelial dysfunction, a marker of atherosclerosis, is independent of metabolic syndrome in NAFLD patients. Int. J. Hepatol..

[76] Akabame S., Hamaguchi M., Tomiyasu K.-I., Tanaka M., Kobayashi-Takenaka Y., Nakano K., Oda Y., Yoshikawa T. (2007). Evaluation of vulnerable coronary plaques and non-alcoholic fatty liver disease (NAFLD) by 64-detector multislice computed tomography (MSCT). Circ. J..

[77] Kasper P., Martin A., Lang S., Kütting F., Goeser T., Demir M., Steffen H.-M. (2020). NAFLD and cardiovascular diseases: A clinical review. Clin. Res. Cardiol..

[78] Keskin M., Hayıroglu M.I., Uzun A.O., Guvenç T.S., Sahin S., Kozan O. (2017). Effect of nonalcoholic fatty liver disease on in-hospital and long-term outcomes in patients with ST-segment elevation myocardial infarction. Am. J. Cardiol..

[79] Bonapace S., Valbusa F., Bertolini L., Pichiri I., Mantovani A., Rossi A., Zenari L., Barbieri E., Targher G. (2014). Nonalcoholic fatty liver disease is associated with aortic valve sclerosis in patients with type 2 diabetes mellitus. PLoS ONE.

[80] Targher G., Byrne C.D., Tilg H. (2020). NAFLD and increased risk of cardiovascular disease: Clinical associations, pathophysiological mechanisms and pharmacological implications. Gut.

[81] Targher G., Valbusa F., Bonapace S., Bertolini L., Zenari L., Pichiri I., Mantovani A., Zoppini G., Bonora E., Barbieri E. (2014). Association of nonalcoholic fatty liver disease with QTc interval in patients with type 2 diabetes. Nutr. Metab. Cardiovasc. Dis..

[82] Targher G., Valbusa F., Bonapace S., Bertolini L., Zenari L., Rodella S., Zoppini G., Mantovani W., Barbieri E., Byrne C.D. (2013). Non-alcoholic fatty liver disease is associated with an increased incidence of atrial fibrillation in patients with type 2 diabetes. PLoS ONE.

[83] Karajamaki A.J., Patsi O.P., Savolainen M., Kesaniemi Y.A., Huikuri H., Ukkola O. (2015). Non-alcoholic fatty liver disease as a predictor of atrial brillation in middle-aged population (OPERA study). PLoS ONE.

[84] Mantovani A., Rigamonti A., Bonapace S., Bolzan B., Pernigo M., Morani G., Franceschini L., Bergamini C., Bertolini L., Valbusa F. (2016). Nonalcoholic fatty liver disease is associated with ventricular arrhythmias in patients with type 2 diabetes referred for clinically indicated 24-hour holter monitoring. Diabetes Care.

[85] Carnagarin R., Matthews V., Zaldivia M.T.K., Peter K., Schlaich M.P. (2019). The bidirectional interaction between the sympathetic nervous system and immune mechanisms in the pathogenesis of hypertension. Br. J. Pharmacol..

[86] Zhao Y.-C., Zhao G.-J., Chen Z., She Z.-G., Cai J., Li H. (2020). Nonalcoholic fatty liver disease. Hypertension.

[87] Abbdullahi A.M., Isah U.A. (2025). Stroke risk among Nigerians with diabetes and hypertension: A pilot retrospective cohort study. Brain Heart.

[88] Huh J.H., Ahn S.V., Koh S.B., Choi E., Kim J.Y., Sung K.-C., Kim E.J., Jeong Bae Park J.B. (2015). A prospective study of fatty liver index and incident hypertension: The KoGESARIRANG study. PLoS ONE.

[89] Lau K., Lorbeer R., Haring R., Schmidt C.O., Wallaschofski H., Nauck M., John U., Baumeister S.E., Völzke H. (2010). The association between fatty liver disease and blood pressure in a population-based prospective longitudinal study. J. Hypertens..

[90] Lorbeer R., Bayerl C., Auweter S., Rospleszcz S., Lieb W., Meisinger C., Heier M., Peters A., Bamberg F., Hetterich H. (2017). Association between MRI-derived hepatic fat fraction and blood pressure in participants without history of cardiovascular disease. J. Hypertens..

[91] Ryoo J.-H., Suh Y.J., Shin H.C., Cho Y.K., Choi J.-M., Park S.K. (2014). Clinical association between non-alcoholic fatty liver disease and the development of hypertension. J. Gastroenterol. Hepatol..

